# A Real-Time High Performance Computation Architecture for Multiple Moving Target Tracking Based on Wide-Area Motion Imagery via Cloud and Graphic Processing Units

**DOI:** 10.3390/s17020356

**Published:** 2017-02-12

**Authors:** Kui Liu, Sixiao Wei, Zhijiang Chen, Bin Jia, Genshe Chen, Haibin Ling, Carolyn Sheaff, Erik Blasch

**Affiliations:** 1 Intelligent Fusion Technology, Inc., Germantown, MD 20876, USA; kui.liu@intfusiontech.com (K.L.); sixiao.wei@intfusiontech.com (S.W.); zhijiang.chen@intfusiontech.com (Z.C.); bin.jia@intfusiontech.com (B.J.); gchen@intfusiontech.com (G.C.); 2 Department of Computer and Information Sciences, Temple University, Philadelphia, PA 19122, USA; hbling@temple.edu; 3 Air Force Research Laboratory, Information Directorate, Rome, NY 13441, USA; carolyn.sheaff@us.af.mil

**Keywords:** high performance computation, cloud infrastructure, target detection, target tracking

## Abstract

This paper presents the first attempt at combining Cloud with Graphic Processing Units (GPUs) in a complementary manner within the framework of a real-time high performance computation architecture for the application of detecting and tracking multiple moving targets based on Wide Area Motion Imagery (WAMI). More specifically, the GPU and Cloud Moving Target Tracking (GC-MTT) system applied a front-end web based server to perform the interaction with Hadoop and highly parallelized computation functions based on the Compute Unified Device Architecture (CUDA©). The introduced multiple moving target detection and tracking method can be extended to other applications such as pedestrian tracking, group tracking, and Patterns of Life (PoL) analysis. The cloud and GPUs based computing provides an efficient real-time target recognition and tracking approach as compared to methods when the work flow is applied using only central processing units (CPUs). The simultaneous tracking and recognition results demonstrate that a GC-MTT based approach provides drastically improved tracking with low frame rates over realistic conditions.

## 1. Introduction

Currently, various sensor platforms are used for persistently monitoring very large areas. For instance, Wide Area Motion Imagery (WAMI) systems on aerial platforms flying at 7000 feet and covering an area of a 2 mile radius can be used as an aid in disaster relief, emergency response, and traffic management [1]. Such systems typically produce an overwhelmingly large amount of information as provided in the Columbus Large Image Format (CLIF) dataset [2]. Thus, the management of WAMI is a Big Data problem [3,4]. Monitoring such a large amount of data with a human operator is not feasible, which calls for an automated method of processing the data to support information fusion. The detection and tracking of the moving objects, as well as data storage and retrieval, are critical in the design and implementation of such WAMI data management systems. Compared with traditional video surveillance tasks, WAMI collections are characterized by large areas, low frame rates, while the pixels on targets are small. Thus traditional computer vision methods or architectures are inadequate to solve these problems due to the high computational complexity and low resolution of the target of interest in the imagery. This paper presents (1) combines Cloud technology with Graphic Processing Units (GPUs) in a complementary manner within the framework of real-time high performance computation architecture and (2) applies to the application of detecting and tracking multiple moving targets based on Wide Area Motion Imagery (WAMI).

The Cloud computation system gives a flexible architecture which enables applications to add to or modify the data system as the needs change. Cost-effective and readily-available components from any Information Technology (IT) vendors can be used. A Graphical Processing Unit (GPU), found on video cards and as part of display systems, is a specialized processor that can rapidly execute commands for manipulating and displaying images. GPU-accelerated computing offers faster performance across a broad range of designs, animations and video applications. This paper presents a design and implementation of a computation architecture integrating the Cloud and GPU high efficiency platforms for detecting MUltiple MOving Targets (MUMOTs) based on WAMI images. Using the resulting detections and tracks, it is possible to implement a pattern-of-life (PoL) analysis [5,6] and anomaly detection algorithms for MUMOTs. However, it is worth noting that the introduced approach in this paper is general purpose in the sense that it is applicable to other High Performance Computation (HPC) problems such as statistical big data analytics based on many object features such as size, frequency and moving direction [7], aerospace satellite orbit propagation problems [8,9] and pedestrian detection [10,11] in large scale images. Moreover, the same computation architecture can be applied to conventional computer vision problems. The illustration of the speed-up performance on the WAMI dataset is demonstrated in the experiment section (Section 4).

**Figure 1:**
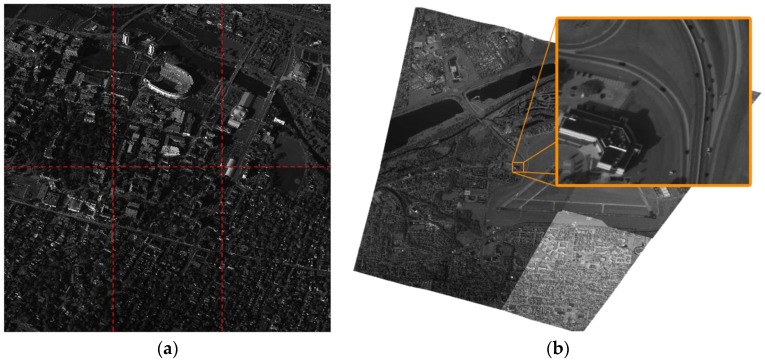
(**a**) Example images from the AFRL CLIF dataset [1] and (**b**) the WPAFB dataset [12]. The CLIF images are not stitched and the dashed red lines show the boundary of six images from different electro-optical (EO) cameras; whereas the WPAFB images are the results after stitching the image from six cameras.

Traditional visual detection and tracking algorithms mainly focus on detecting a limited number of objects in small scenes and high frame rate image sequences. Therefore traditional methods cannot be directly generalized to WAMI scenarios with low frame rates. The large scale images taken by WAMI systems are more than 140,000,000 pixels as shown in Figure 1. The frame rate of the real-time input image is at most two frames per second. Objects in WAMI data are much smaller the full motion imaging systems, with vehicle sizes ranging from 4 to 70 pixels in grayscale image groups. The lack of computationally efficient analysis tools has become a bottleneck for utilizing WAMI data in urban surveillance. Accordingly, it is desirable to develop a computational and data management platform for detecting multiple moving objects based on large scale aerial images via high performance computing technology.

Target tracking has been extensively investigated as described in many papers [13,14,15,16,17]. Santhaseelan et al. [18] proposed a robust feature-based method to track objects in WAMI data by exploiting local phased and orientation information based on the monogenic signal representation. However, this method does not present a systematical architecture for a prototype. Eekeren et al. [19] built a pipeline which comprises a static vehicle detector, a smart object filtering algorithm using a 3D building reconstruction, and multi-camera object tracking based on template matching. This method focused on 3D reconstruction and abnormal detection while no experimental results are reported. Pelapur et al. [20] proposed Likelihood of Features Tracking (LoFT) system that is based on fusing multiple sources of information about the target and its environment akin to a track-before-detect (TBD) approach. These proposed methods reported on track accuracy versus comparisons of the computational complexity and performance time that is dependent on actual real-time image registration and stitching methods. Likewise, these proposed methods or systems did not highlight the MUMOTs problem under realistic conditions. The proposed computation architecture presented in this paper differs from all the previous works in the sense that this architecture makes the inevitable high computational complexity problem a real-time implementation by using Cloud architecture.

This paper develops a framework which utilizes image sets from WAMI sensor streaming to detect and track objects of interest for real-time applications. The High Performance Computation (HPC) framework includes: An Apache Hadoop distributed storage and distributed processing system; Multiple GPUs for parallel computations; and a front-end web server for data storage and retrieving. The Apache Hadoop framework utilizes the MapReduce programming model to distribute the computational algorithms parallel on each cluster. Each cluster includes a CPU and multiple GPUs. The MapReduce program is composed of a Map() procedure which performs registration, background generation, foreground generation, vehicle detection, data association and trajectories generation and a Reduce() procedure which performs a summary operation (generating the target track identifications (IDs) and saving the detection and trajectories information in HDFS (Hadoop Distributed File System)). Moreover; the MapReduce program arranges the distributed clusters and runs the GPU tasks in a Compute Unified Device Architecture (CUDA) parallel computing platform. In this GC-MTT MUMOTs detection and tracking system, registration, background generation and foreground generation are performed in GPUs. A front-end web server is developed to present the most useful data and obtain abstract and meaningful information for human analysts. Specifically, a web-based data visualization system is developed to communicate with the Apache Hadoop cluster for conducting the real-time tracking analysis and user interaction. More details is introduced in Section 3.

Comparing the MUMOTs tasks with CPUs or GPUs alone, the application of distributed and parallel computing structure based on Apache Hadoop MapReduce and CUDA Basic Linear Algebra Subroutines (cuBLAS) can achieve a real-time outcome of detection and tracking [21,22]. Moreover, the obtained detection and recognition results for the MUMOTs indicate that the parallel-based approach provides drastically improved, speed-up performance in real-time and under realistic conditions. One of the contributions of the paper is that a non real-time algorithm achieves real-time performance based on the application of a Cloud and GPU parallel computing infrastructure.

Cloud and parallel computation has become increasingly important for computer vision and image processing systems [23,24]. A Cloud-based framework uses Cloud computing, which is constructed within high performance clusters to include the combination of CPUs and GPUs [25,26,27]. A local server in the Cloud is provided for the data storage and retrieving and a web portal server is provided for the user. Based on the local server, the tracking results (trajectories of the objects of interest) generated from the computation nodes in a Hadoop Distributed File System (HDFS) are converted and saved in the data base. From the web portal, the user chooses algorithms, datasets and system parameters such as the number of computation nodes in operation, the image registration methods and the processing units (with or without Hadoop, CPU or GPU processing). A controller in the Cloud will then decide the amount of computing resources to be allocated to the task in order to achieve the user's requirements of performance. Inside the Cloud, each computation node is actually within each cluster. One CPU and multiple GPUs comprise each cluster. The computation nodes are capable of running various high performance computation tasks. In this paper, the high performance tasks for image-based MTT include registration, background generation, foreground generation, detection, data association, and trajectories generation which are usually run by several threads in one or more computation nodes in parallel.

The rest of the paper is as follows. Section 2 summarizes the GC-MTT framework. Section 3 discusses the Cloud and GPU infrastructure, block-wise image registration, parallel implementation and the front-end web-based demonstration. Section 4 presents the detection and tracking results and the speed-up performance and Section 5 provides conclusions.

## 2. Overview of the Proposed GC-MTT Framework

**Figure 2:**
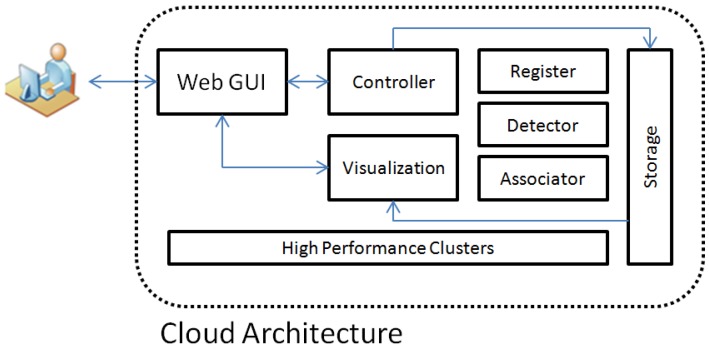
The high level view of the Cloud system for MUMOTs detection and tracking.

Until recently, typical experiments conducted for WAMI object tracking reported in the literature mostly are CPU-based schemes that do not explicitly consider computational complexity. However, in practice, the resolution of a WAMI dataset captured under realistic conditions is considerably large while the resolution of objects of interest is small. As a result, target detection, recognition, and tracking becomes quite challenging where many false alarms are generated by existing approaches. Key tracking computation modules consist of a register, detector and associator in the Cloud architecture. Figure 2 depicts a high level view of a host in the Cloud system. The HPC serves as the computation nodes in the Cloud. All components of the same task have access to share storage in the Cloud in the HDFS. The user only interacts with the system through the Web Graphic User Interface (GUI). The user’s computing requests from the web GUI are passed to the controllers for further processing. The controller assigns an appropriate number of jobs to computation nodes for each request. Each node runs one assigned task (Register, Detector and Associator) and sends the results back to HDFS and then the local server. The web GUI will then display the processing results in real-time once the backend processing finishes. The local server uses a database to store real-time performance of all tasks in the system. It can also monitor the Cloud’s performance such as average CPU/GPU load and memory usage [28]. The user can choose what metrics to be displayed on the web GUI and can call other visual analytic tools such as the Global Positioning System (GPS) coordinates of the objects of interest at a particular instant, the 3-dimensional trajectories of an object [29], or pattern of life (PoL) analysis of MUMOTs. In the MUMOTs detection and tracking scenarios, the key components of the proposed Cloud and GPU system perform the following tasks:

- *User*: The user chooses the system configuration, such as the options of various register, detector and associator algorithms; assigns computation nodes in operation and the selection of processing units, and sends comments to the system to initiate a task.
- *Web GUI*: The web GUI communicates with the user and by receiving input commands, displaying processing results and presenting analytical system performance.
- *Controller*: The controller receives commands from the web GUI, makes decisions on how many resources are needed to satisfy the required performances, assign jobs and tasks to computation nodes in the Cloud, calculates processing speed in real-time, and informs the web GUI the processing results.
- *Visualization*: Local server collects performance metrics such as processing speed and system load, and provides a web GUI query service when there is a need to display the metrics.
- *High performance clusters*: Each cluster can act as a register, detector or associator in the system. The tasks which will be performed in CPU or GPUs are decided by the controller.

Details of the above key modules will be further described in the following sections.

## 3. Proposed MUMOTs Detection Infrastructure

### 3.1. Detail Cloud Computation Infrastructure

**Figure 3:**
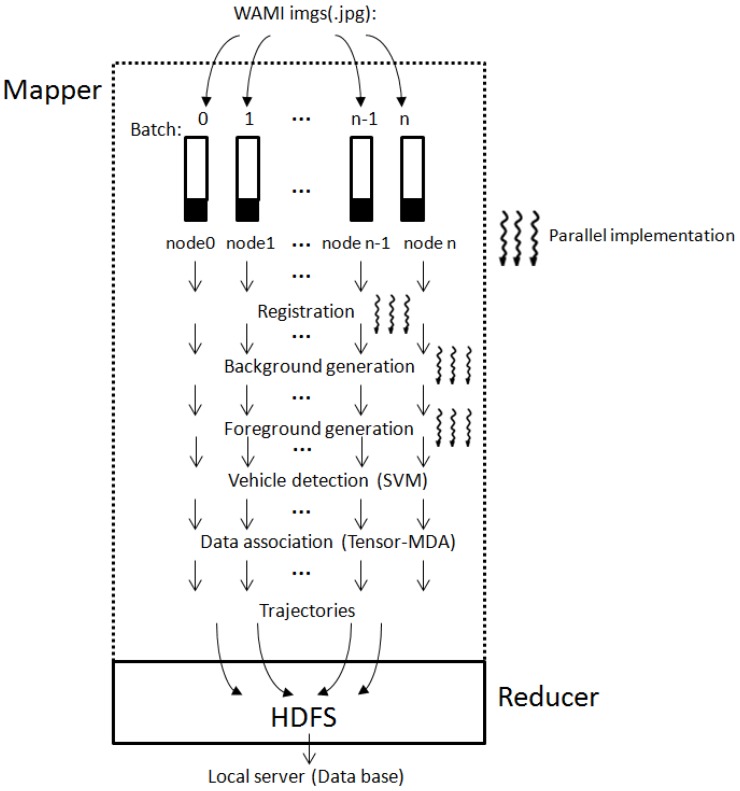
The Cloud and GPU high performance computation infrastructure for MUMOTs problem.

Figure 3 shows the overall data flow of the Cloud and GPU infrastructure. Besides GPU acceleration, all the WAMI image processing tasks perform registration, background and foreground generation, while vehicle detection and data association were performed on a Cloud using GPU enabled computation nodes.

This Cloud computation system consists of the following components:

#### 3.1.1. Mapper in Hadoop

The Mapper of the GC-MTT system starts with user's selection of the WAMI image dataset. The Controller will automatically distribute the images to the computation nodes. A *master node* (could be node0) populates the jobs into the computation nodes and launches a number of operational nodes. Based on each operational computation node, the image registration transforms different sets of data into one coordinate system, since the homography matrix generated from the image registration can further achieve the rotation and translation matrices. With the rotation and translation matrices, the coordinate in the previous frames are projected in to the current frames and thus a general background of the image sets can be generated through the image stitching techniques.

The *background generation* process includes a background setting step, an image averaging step, and a background extraction step. The background extraction is a parallelized process implemented based on the GPU which uses data structure *dim3*.

The *Foreground generation* process comprises a pixel value comparison step, a value assigning step, and a foreground extraction step. It also implements the Hyper-Q computation framework to enable multiple CPU cores to launch a job on a single GPU simultaneously for increasing GPUs utilization, minimizing CPU idle time, and introducing a Grid Management Unit (GMU) to create multiple hardware work queues to reduce synchronization time [30].

The *SVM (Support Vector Machine) classification* process implements histogram of oriented gradients (HoG) to compute color gradients and obtain gradient magnitudes and orients via convolution, and then calculates probabilities or confidence levels of the MUMOTs based on the gradient magnitudes and orientations.

The *Data association process* is the key component to combine the detected MUMOTs in the consecutive WAMI frames into target trajectories. Details of the data association process can be found in our previous work in [31].

#### 3.1.2. Reducer in Hadoop

The Reducer in the Hadoop system performs a summary operation which generates the target track IDs and saves the detection and trajectories information in HDFS.

#### 3.1.3. System Implementation

**Figure 4:**
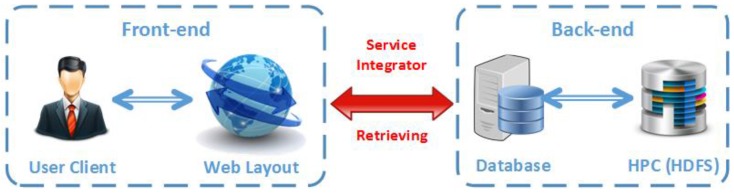
System Implementation Architecture.

A distributed, multi-node Apache Hadoop cluster was designed in the back-end for conducting the real-time computation analysis, which includes a Hadoop Distributed File System (HDFS) based on HPCs running on Ubuntu Linux. In the front-end, a web-based data visualization system presents the most useful data and obtains meaningful information for human analysts in support of high level information fusion [32] and context-enhance information fusion [33]. Figure 4 details the system architecture of both the front-end and the back-end systems.

On the back-end, the Hadoop is the main framework which implements all the tracking algorithms. Specifically, Hadoop is a framework for job scheduling and cluster resource management and MapReduce is system based parallel processing of large data sets. Once Hadoop is running, a master machine will assign computation works towards different slaver machines, and then all the outputs from slaver machines will be collected and stored in the HDFS, which provides high-throughput access to application data. The service integrators will perform the interaction between front-end and back-end and provide analyzed real-time data to the front-end database for user requests.

On the front-end, the main components are Client and Web Server. The Client is directly connected to the Web Server via Cache. When any functions are required by client, the web server will call their related application programming interface (API) to create information requests to the supported database. Once user requests are sent out from front-end web GUI, they will be accepted by the service distributor. Then multi-threading and multi-stream based processing will be triggered to execute and generate tracking results by service integrators. Finally, the demonstration results will be sent back to front-end web-based display for the user interaction.

### 3.2. Block-Wise Image Registration

Due to characteristics of WAMI images, including the overwhelming increase in image size, results present a prohibitive memory requirement and computational complexity such that the coarse image registration usually takes an unfavorable long processing time based on CPU infrastructure.

**Figure 5:**
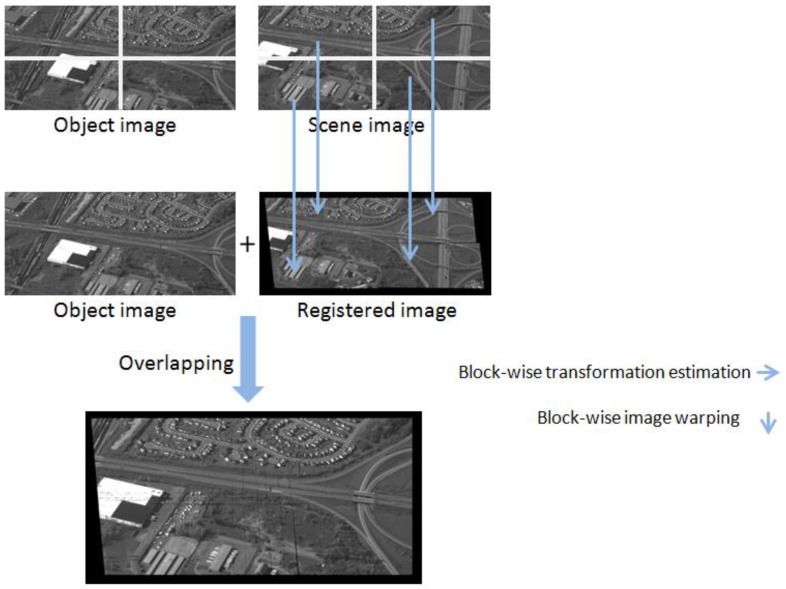
The concept of block-wise image registration.

A fast block-wise registration process, shown in Figure 5, is proposed via a CUDA based parallel computing infrastructure, which comprises:

- a block-wise Speed Up Robust Feature (SURF) [34] extraction process for each image partition;
- a point matching process for each image partition;
- a random sample consensus (RANSAC) algorithm [35] to remove outlier points from the image partitions; and
- a transformation estimation process of the image partition to generate block-wise homography matrices.

Each registration kernel is configured to have one computation node integrated with *n* groups of four image partitions at a time instant. Stitching portions of the registered image partitions is based on the block-wise homography matrices generated from the transformation estimation process, wherein a number of threads per block is consistent with available shared memory of the GPUs.

### 3.3. Parallel Background and Foreground Generation

**Figure 6:**
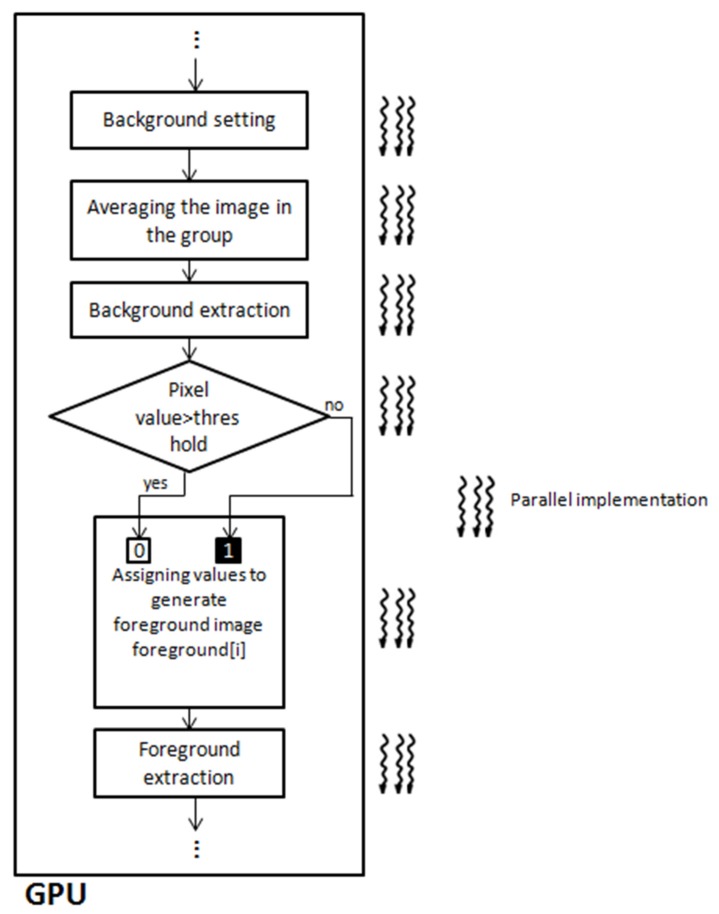
The concept of GPU based background and foreground generation.

As it can be seen from Figure 6, a GPU-based background and foreground generation is provided. As well known, CPU-based image processing techniques requires many two-dimensional traversals of the image sequences. This 2D operational structure includes many computations, especially the input sequence is a set of large size images.

The following steps are performed in GPUs: (1) Background setting of each image with a partition to mask with zero pixel values; (2) Image averaging and background extraction are then performed; (3) To generate foreground, pixel values of output images can be compared with a predetermined threshold value. For example, if a gray value of a pixel is larger than the predetermined threshold, the pixel can be determined as a portion of the foreground image, and the pixel can be assigned as a value of “0”. On the other hand, if a gray value of a pixel is smaller than the predetermined threshold value, the pixel can be determined as a portion of the background image, and the pixel can be assigned as a value of “1”. Thus the binary foreground image is extracted.

### 3.4. Front-End Web-Based Demonstration

To present the most useful data and obtain abstract and meaningful information for human analysts [32], a data visualization system is introduced, which applies a front-end web based server to perform the interaction with Hadoop and highly parallelized computation functions based on CUDA. The following are some essential components of establishing a usable interface:

- Programming Languages: PHP, JavaScript, HTML, CSS;
- Webserver: Node.js;
- Database: MongoDB;
- Platform: Cloud 9 IDE; and
- Plugin: Bootstrap, JQuery

To distinguish between multiple targets being tracked in an image, a tensor-based data association method is employed to disambiguate different targets for the user.

### 3.5. Tensor Based Multi-Target Data Association

In this section, a brief introduction is provided about tensor and its rank-1 approximation. Details about the tensor formulation and data association can be found in [31]. A tensor is the high dimensional generalization of a matrix. For a *K*-order tensor $S\in {\mathbb{R}}^{I_{1}\times I_{2}\times \ldots \times I_{K}}$, each element is represented as $s_{i_{1}\ldots i_{k}\ldots i_{K}}$ and $1\leq i_{k}\leq I_{k}$. In the tensor terminology, each dimension of a tensor is associated with a mode. Like matrix-vector and matrix-matrix multiplication, tensor has similar operation, we give the following definition: the n-mode product of a tensor $S\in {\mathbb{R}}^{I_{1}\times \ldots I_{n-1}I_{n}\ldots \times I_{K}}$ and a matrix $E\in {\mathbb{R}}^{I_{n}\times J_{n}}$, denoted by $S{⊗}_{n}E$, is a new tensor $B\in {\mathbb{R}}^{I_{1}\times \ldots I_{n-1}J_{n}\ldots \times I_{K}}$. The notation is represented as: $B=S{⊗}_{n}E$, $b_{i_{1}\ldots i_{n-1}j_{n}i_{n+1}\ldots i_{K}}=\sum_{i_{n}=1}^{I_{n}}s_{i_{1}\ldots i_{n-1}i_{n}\ldots i_{K}}e_{i_{n}j_{n}}$. In particular, the *n*-mode product of *S* and a vector $\Pi \in {\mathbb{R}}^{I_{n}\times 1}$, denoted by $S{⊗}_{n}\Pi$, is the *K* − 1 order tensor ${\left(S{⊗}_{n}\Pi \right)}_{i_{1}\ldots i_{n-1}i_{n+1}\ldots i_{K}}=\sum_{i_{n}=1}^{I_{n}}s_{i_{1}\ldots i_{n-1}i_{n}\ldots i_{K}}\pi_{i_{n}}$.

The framework is outlined in the Algorithm 1. Generally, multi-target association is performed with the batch way. When *K* frame observations are available, association hypotheses (trajectories) are generated first. With all these hypotheses, a tensor is constructed by computing the trajectory affinities. Then, the $\ell_{1}$ norm tensor power solution is performed. Algorithm 1 presents the procedures for the tensor based multi-target association.

**Algorithm 1.** Tensor based multi-target association1: Input. *M* frame observation sequence. $\;t_{0}$: Start frame, *K*: Number of a batch frames. 2: Output: target associations 3: **while**
$t_{0}+K-1\leq M$
**do** 4:   Collect a batch of K frames observation   $\Phi =\left\{O\left(t_{0}\right),\;O\left(t_{0}+1\right),\ldots ,O\left(t_{0}+K-1\right)\right\}$5:  Generate two-frame association hypotheses. 6:  Generate global trajectory hypotheses. 7:  Compute the trajectory affinities and construct the *K* − 1 order tensor *S*. 8:  Initialize the approximate vectors. 9:  $\ell_{1}$ row/column unit norm tensor power iteration. 10:   **Solution**: Discretize the approximate vectors. 11:   $t_{0}\leftarrow t_{0}+K-1$ 12: **end while**

## 4. Experiments and Results

**Figure 7:**
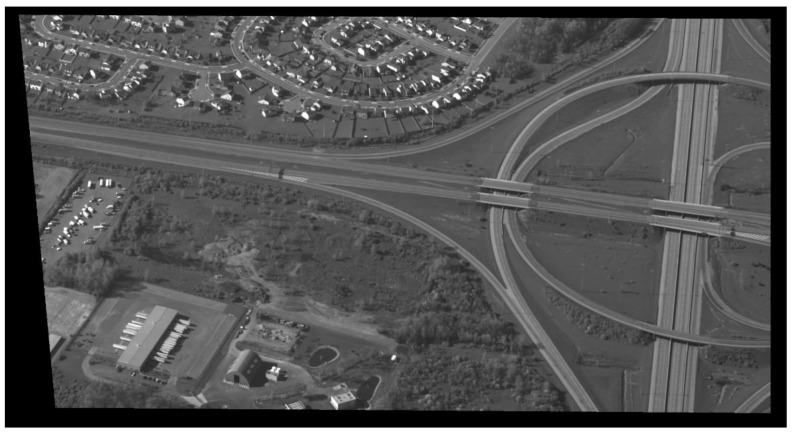
An example of extracted background.

**Figure 8:**
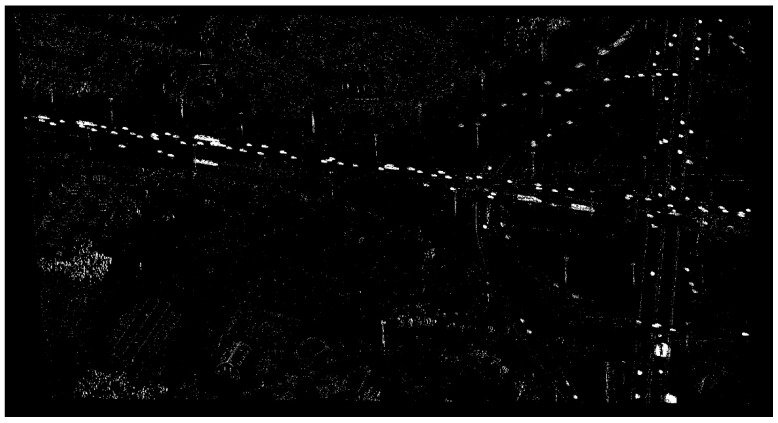
An example of extracted foreground.

**Figure 9:**
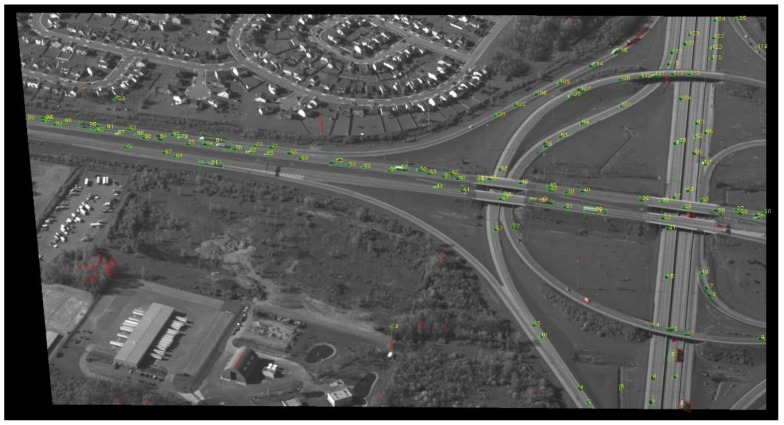
An example of MUMOT detection.

**Figure 10:**
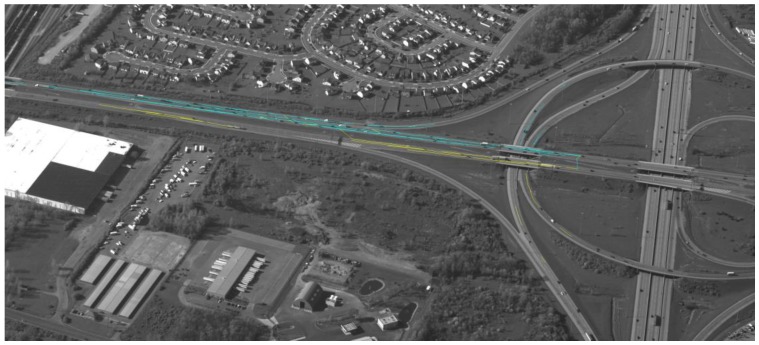
An example of target trajectories generation.

Figure 7, Figure 8, Figure 9 and Figure 10 show examples of background generation, foreground generation, MUMOTs detection and trajectories generation; respectively.

The recently available GPU NVidia Kepler K20Xm with 2688 CUDA cores and 6144 MB Memory was applied on each cluster. In this work, the number of operational computation nodes is 4. The platform of CPU is Intel Xeon E5-2630 Processor. Another system used was the Ubuntu 14.04, OpenCV 2.4.13 and CUDA 7.5. The MUMOT detection and tracking algorithms were implemented in these two systems for comparison, while other implementations have supported investigations in cognitive radio [36] and long-range communications [37]. The dataset used to verify the speedup performance is from dataset Columbus Large Image Format 2006 [2] where the size of airborne image frame is 2672 × 1200 pixels.

**Figure 11:**
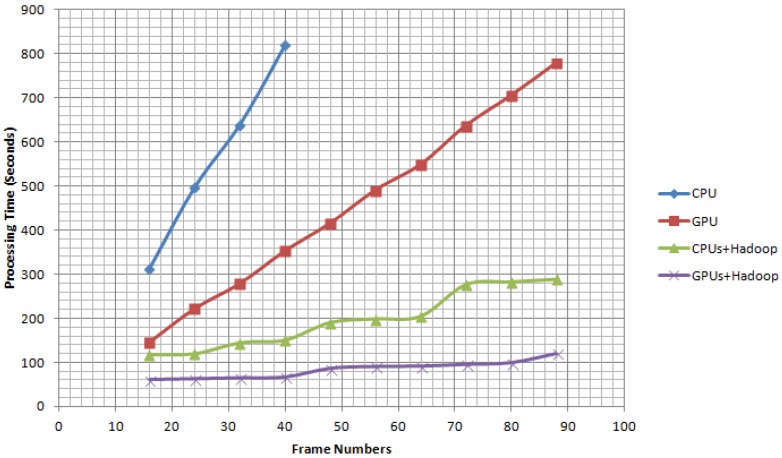
Computation performance comparison based on CPU, GPU, Hadoop + CPUs and Hadoop + GPUs.

Figure 11 shows the processing time for the algorithm applied on different numbers of frames. 11 × 8 (88 frames) WAMI images were used to test the computation performance of the GC-MTT computation architecture. The run time is generated based on the four different computation architectures (CPU, GPU, Hadoop + CPUs and Hadoop + GPUs); respectively. When processing 56 frames, the duration of Hadoop + CPUs was 192 s. The frame rate of this architecture is about 0.29 frames per second (fps). While based on the computation architecture Hadoop + GPUs, the speed-up performance is more obvious. The duration of processing was 90.94 s. The frame rate of the architecture Hadoop + GPUs is 0.61 frames per second. It is worth noting that the computation time is 1206 s based on CPU and 500 s based on GPU without the Hadoop and the same input, which is to say, the value of Hadoop implementation provides 4 times improvement of the computation time (the number of computation node is 4). As can be expected, the overhead would be much smaller when the input of images is more than 100 frames. In the CPU and GPU without Hadoop versions, the run time increases when the frame numbers were larger. In the versions with Hadoop implementation, it was observed that it does not significantly change.

**Figure 12:**
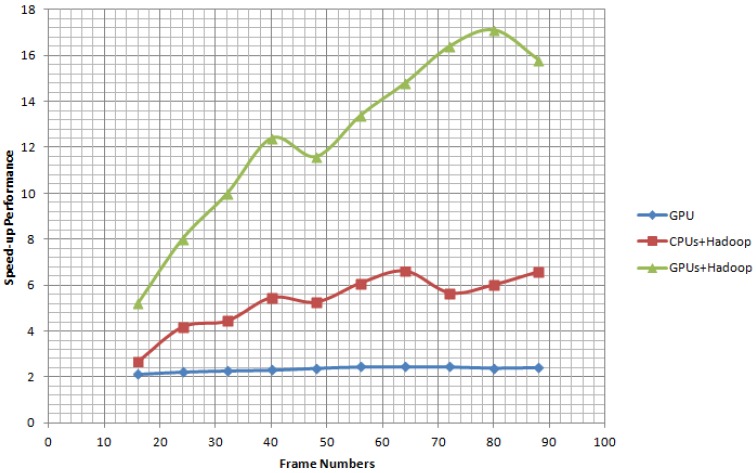
Speed-up performance of GPU, Hadoop + CPUs and Hadoop + GPUs comparing to CPU platform.

Figure 12 shows the speed-up performance of the platforms GPU, Hadoop + CPUs and Hadoop + GPUs as compared to the CPU platform. As can be seen, the speed-up performance of platforms Hadoop + CPUs achieves 4 times increase in fps at frame number 24. The speed-up performance of the platform Hadoop + GPUs provides very promising results from the very beginning. With the increasing of the frame number, the speed-up performance improves greatly. The speed-up performance of GPU platform stabilizes around 2 fps. This result further demonstrates that the proposed GPUs and Hadoop integration architecture is a highly efficient computation platform.

**Figure 13:**
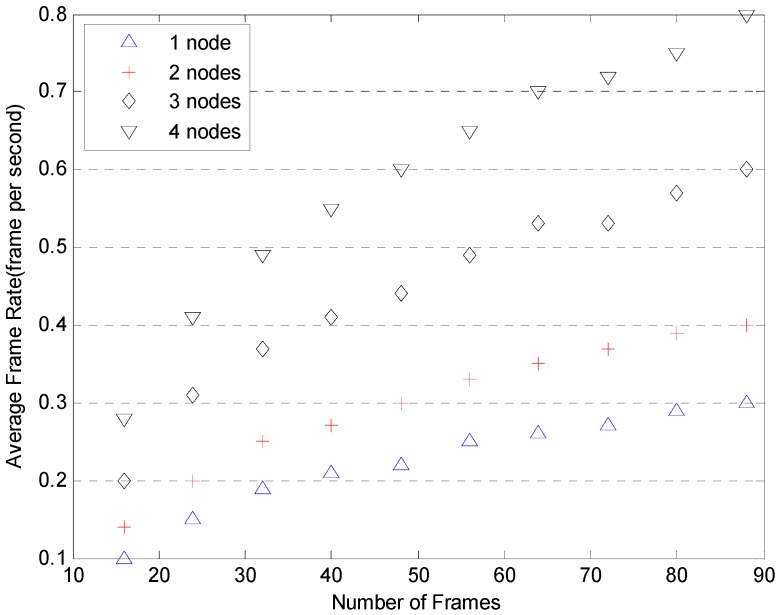
Average frame rate for multiple nodes based on GPUs.

**Figure 14:**
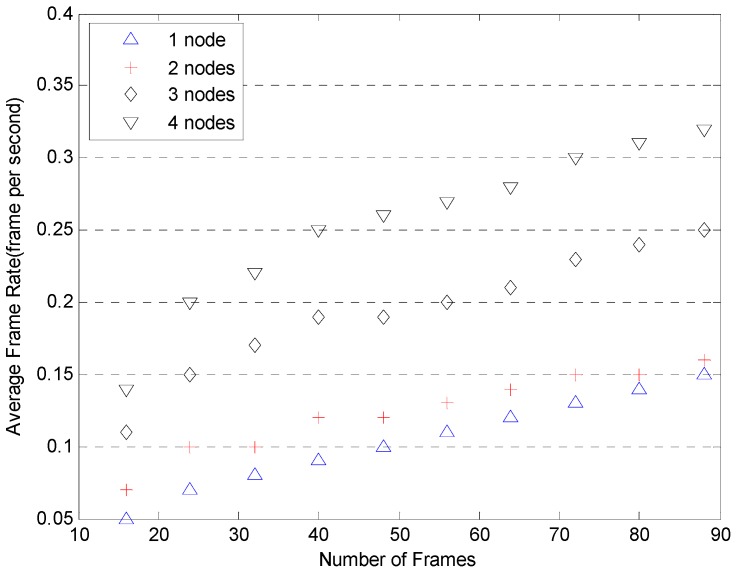
Average frame rate for multiple nodes based on CPUs.

**Figure 15:**
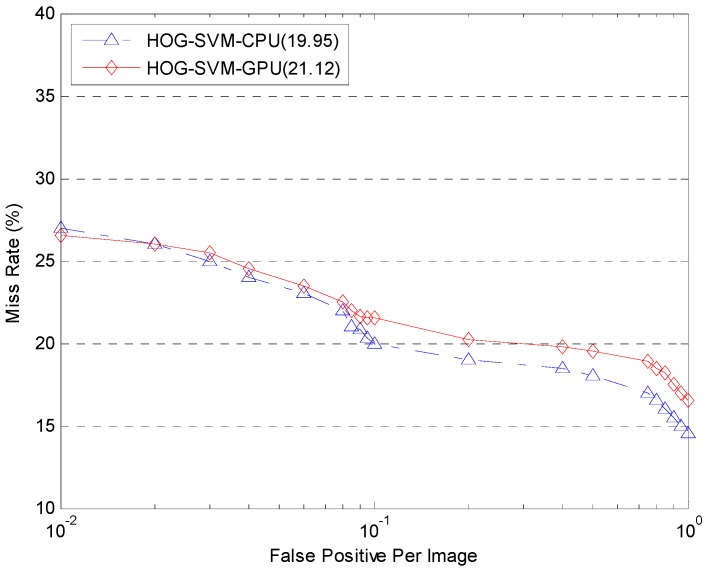
Objective term (lower is better).

More tests illustrating the speed advantages of Cloud are shown in Figure 13 and Figure 14. The tests were conducted with two, three and four computation nodes available to the framework. The number of frames involved in each setup varies from 8 up to 90 frames. The frame rates are plotted against the number of frames for each setup based in GPUs and CPUs in Figure 13 and Figure 14. The results for the single node setup are included for comparison. As can be seen in Figure 13 and Figure 14, with the implementation of a Cloud architecture, the average frame rates are improved as expected whether using the GPU or CPU platform. Figure 15 illustrates the detection miss rate (non-detected cases) as a function of the false positive per image (FPPI)—such as a window wrongly classified as pedestrians. The detection performance of CPU and GPU is similar. As the FPPI increases, the miss rate decreases, leading to a more reliable system. The area below the curves is shown as legend values: the lower the value, the more reliable is the detector [38]. As can be seen in Figure 15, the performance of CPU and GPU shows very little difference.

## 5. Conclusions

In this paper, a Cloud and GPU-based high performance computation system for MUltiple MOving Targets (MUMOTs) detection and tracking was introduced. The proposed GPU-based and Cloud Multiple Target Tracking (GC-MTT) method is one of the first attempts at detecting and tracking MUMOTs from WAMI image sequences using Cloud and parallel computation technology. The proposed GC-MTT computation architecture for MUMOTs detection and tracking consists of registration, background generation, foreground generation, vehicle detection, data association and trajectories generation. It was shown that by applying the GC-MTT method leads to a much faster, more reliable, and real-time performance of detection and tracking as compared to situations when the workflow is applied on a single CPU or GPU alone. Future work will include pattern of life (PoL) trajectory analysis comparison with cloud architectures for documenting and understanding multiple interacting target routines in urban areas such as that for traffic analysis. This information can then be potentially used to build models of normalcy, predict future actions, and determine anomalous behaviors for urban safety and intelligent highways.
